# KLF9 and KLF13 transcription factors boost myelin gene expression in oligodendrocytes as partners of SOX10 and MYRF

**DOI:** 10.1093/nar/gkac953

**Published:** 2022-11-01

**Authors:** Celine Bernhardt, Elisabeth Sock, Franziska Fröb, Simone Hillgärtner, Mona Nemer, Michael Wegner

**Affiliations:** Institut für Biochemie, Friedrich-Alexander-Universität Erlangen-Nürnberg, Erlangen, Germany; Institut für Biochemie, Friedrich-Alexander-Universität Erlangen-Nürnberg, Erlangen, Germany; Institut für Biochemie, Friedrich-Alexander-Universität Erlangen-Nürnberg, Erlangen, Germany; Institut für Biochemie, Friedrich-Alexander-Universität Erlangen-Nürnberg, Erlangen, Germany; Molecular Genetics and Cardiac Regeneration Laboratory, Department of Biochemistry, Microbiology and Immunology, University of Ottawa, Ottawa, Canada; Institut für Biochemie, Friedrich-Alexander-Universität Erlangen-Nürnberg, Erlangen, Germany

## Abstract

Differentiated oligodendrocytes produce myelin and thereby ensure rapid nerve impulse conduction and efficient information processing in the vertebrate central nervous system. The Krüppel-like transcription factor KLF9 enhances oligodendrocyte differentiation in culture, but appears dispensable in vivo. Its mode of action and role within the oligodendroglial gene regulatory network are unclear. Here we show that KLF9 shares its expression in differentiating oligodendrocytes with the closely related KLF13 protein. Both KLF9 and KLF13 bind to regulatory regions of genes that are important for oligodendrocyte differentiation and equally recognized by the central differentiation promoting transcription factors SOX10 and MYRF. KLF9 and KLF13 physically interact and synergistically activate oligodendrocyte-specific regulatory regions with SOX10 and MYRF. Similar to KLF9, KLF13 promotes differentiation and myelination in primary oligodendroglial cultures. Oligodendrocyte differentiation is also altered in KLF13-deficient mice as demonstrated by a transiently reduced myelin gene expression during the first postnatal week. Considering mouse phenotypes, the similarities in expression pattern and genomic binding and the behaviour in functional assays, KLF9 and KLF13 are important and largely redundant components of the gene regulatory network in charge of oligodendrocyte differentiation and myelination.

## Graphical Abstract

**Graphical Abstract:**
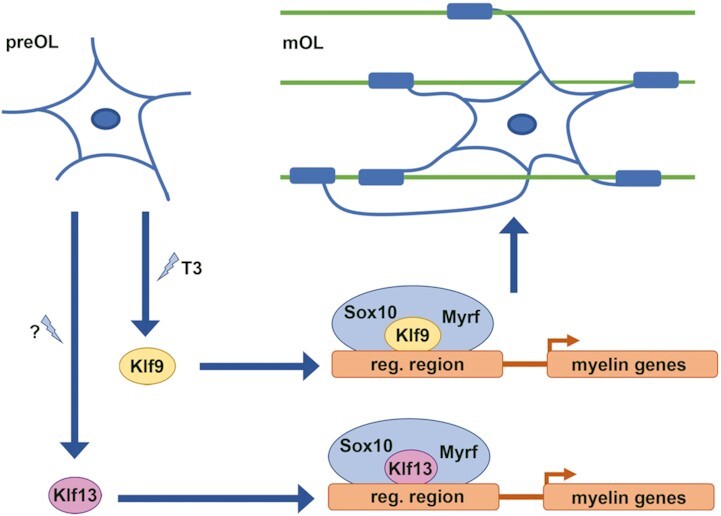
Proposed model for the redundant function of Klf9 and Klf13 transcription factors and synergistic interaction with Sox10 and Myrf in oligodendrocyte differentiation.

## INTRODUCTION

In the vertebrate nervous system, the saltatory mode of nerve conduction helps to speed up the transfer and processing of information. It requires axons to be myelinated, either by oligodendrocytes in the central nervous system (CNS) or by Schwann cells in the peripheral nervous system (PNS). Myelinating oligodendrocytes are the terminally differentiated end point of oligodendroglial development and arise from oligodendrocyte progenitor cells (OPCs) in a strictly regulated process that is controlled both by extrinsic as well as intrinsic factors, the latter being organized in a complex gene regulatory network (1–4). This gene regulatory network contains transcription factors that determine overall levels and changes in gene expression by interacting with each other as well as regulatory RNAs and chromatin modifiers in multiple ways.

The Krüppel-like factor 9 (KLF9) is one such transcription factor. It belongs to the Klf group of zinc finger transcription factors that all contain three neighbouring C2H2 zinc fingers in their carboxyterminal DNA-binding domain (5). Within this group, KLF9 is most closely related to KLF13, KLF14 and KLF16. Together these four factors constitute the BTEB-like subgroup of Klf factors (6,7). In addition to the zinc finger-containing DNA-binding domain, BTEB-like Klf proteins share a nuclear localization signal that immediately precedes the first of the three zinc fingers and an aminoterminal SID domain (5). The SID domain mediates interaction with the Sin3A transcriptional corepressor and overlaps with a poorly characterized transactivation domain (6,7). Identified interaction partners of BTEB-like Klf factors include not only corepressors but also transcriptional coactivators such as p300/CBP and PCAF. Accordingly, BTEB-like Klf proteins appear to function as transcriptional activators or repressors in a context-dependent manner (6,7).

KLF9 has previously been identified as a transcription factor that is strongly induced in differentiating oligodendrocytes by thyroid hormone (T3) and may constitute an important effector downstream of T3 on the transcriptional level (8,9). In agreement with the role of T3 as an extrinsic signal for and timer of oligodendrocyte differentiation (10), KLF9 has been shown to increase myelin gene expression and morphological differentiation of oligodendrocytes in culture (8). However, KLF9-deficient mice exhibited normal CNS myelination, which was attributed to the multiplicity and redundancy of stimuli for oligodendrocyte differentiation during developmental myelination in the early postnatal CNS. So far, it has not been studied how KLF9 fits into the oligodendroglial gene regulatory network and how it may interact on a molecular level with other transcription factors known to impact oligodendrocyte differentiation and myelin gene expression.

Here, we show that KLF9 cooperates with the oligodendroglial lineage determinant and HMG-domain transcription factor SOX10 (11) as well as with MYRF, the central regulator of myelination (12), in the activation of regulatory regions associated with oligodendrocyte differentiation and myelin gene expression. Surprisingly, we found similar activities for KLF13, a closely related transcription factor that also occurs in differentiating oligodendrocytes and binds to oligodendrocyte-specific regulatory regions in vivo. Both gain- and loss-of-function studies in oligodendroglial cultures and the analysis of KLF13-deficient mice support a role of KLF13 during differentiation. This argues for at least partial functional redundancy between KLF9 and KLF13 and sheds further light on the regulation of oligodendrocyte differentiation and the complexities of gene regulatory networks underlying developmental processes.

## MATERIALS AND METHODS

### Cell culture, transfections and transductions

The mouse N2a neuroblastoma and the human HEK293 kidney cell lines were grown in Dulbecco's modified Eagle's medium supplemented with 10% fetal calf serum. N2a and HEK293 cells were obtained from ATCC. Cell line identity was confirmed by PCR. Data on the rat Oln93 cells are from (13).

Rodent primary oligodendroglial cells were obtained from dissociated brain tissue of newborn (P0–P3) mice or Wistar rats of both sexes. Mouse and rat OPC cultures were prepared from mixed glial cultures by subsequent shake-off and microglia depletion (14). For standard proliferation conditions, rat OPCs were seeded on poly-D-ornithine-coated dishes, mouse OPCs on poly-d-lysine and laminin-coated dishes and grown in defined basal medium containing N2 supplement, 10 ng/ml PDGF-AA and 10 ng/ml FGF2. For specific gene deletion, membrane-permeable TAT-Cre protein was added at 0.2 nM to proliferating OPC cultures from mice with floxed alleles three days before differentiation (15,16). For differentiation, growth factor containing basal medium was replaced by SATO medium containing T3 and T4.

Established cell lines were transfected with Superfect reagent for luciferase assays (Qiagen), polyethylenimine for extract preparation and Lipofectamin 2000 (ThermoFisher) for production of retroviruses. Primary oligodendroglial cells were transduced in the OPC stage with retroviruses at a MOI of 0.5–1 or transfected with 20 pM siRNA pools (siKlf9, Dharmacon #M-059033-02-0005; siKlf13, Dharmacon #M-063210-00-0005; si-non-target, Thermo Fischer Scientific #D-001206-14-05) using Lipofectamine 2000 (Thermo Fischer Scientific).

### Plasmids and retroviruses

Expression plasmids for KLF9 and KLF13 were based on pCMV5 and generated by inserting the mouse open reading frame of *Klf9* (position 530–1264 in NM_010638) between HindIII and EcoRI and the mouse open reading frame of *Klf13* (positions 370–1239 in NM_021366.3) between HindIII and XbaI sites of the multiple cloning site, either alone or in-frame behind a myc-tag. Coding sequences were also inserted into pCAG–IRES–GFP (17) to produce KLF9- or KLF13-expressing retroviruses. Expression plasmids for SOX10, OLIG1, OLIG2 and myc-tagged MYRFΔC with pCMV5 backbone have been described before (11,13,18,19). Luciferase reporter plasmids were pGL2 based (Promega) and contained previously identified regulatory regions of the *Gjc2, Mag, Aatk, Mbp* and *Plp1* genes (see also Figure 4A) inserted alone (in case of promoters) or in combination with a β-globin minimal promoter (in case of enhancers) in front of a firefly luciferase coding sequence (13,18,20,21).

### Luciferase assays

For most luciferase reporter assays in N2a neuroblastoma cells, 0.1 μg pCMV5-based expression plasmids were used in combination with 0.2 μg luciferase reporter plasmid on standard 24-well culture plates. In case of KLF13 expression plasmids, ten-fold lower amounts were used for optimal results. However, qualitatively similar results were also obtained with standard amounts. Total pCMV5 amounts were kept constant. All transfections were carried out in triplicates and had comparable transfection efficiencies in the range of 20–30% as determined by co-transfected GFP. At 48 h post transfection, whole cell extracts were prepared and luciferase activities were determined in the presence of luciferin substrate by addition of ATP and detection of chemiluminescence (18).

### Antibodies against KLF9 and KLF13

Antibodies were raised in rabbit, guinea pig, or chicken against peptides that corresponded to amino acids 24–143 of mouse KLF9 and to amino acids 24–143 of mouse KLF13. The peptides were produced in *Escherichia coli* BL21 pLysS bacteria from a pET28b plasmid after IPTG induction and purified from whole bacterial extracts under denaturing conditions by an aminoterminally fused 6xHis-tag (22). Antiserum from the final bleed or antibodies purified from egg yolks were tested for specificity in Western blot, immunoprecipitation as well as immunocytochemical and immunohistochemical applications.

### Extract preparation, protein interaction studies and Western blot

Whole cell lysates were produced from HEK293 cells transfected with pCMV5-SOX10, pCMV5-mycMYRFΔC, pCMV5-mycKLF9, pCMV5-KLF9 or pCMV5-KLF13, from primary oliogdendroglial cultures or from spinal cord tissue of 3-day-old pups as described (19). For immunoprecipitations, HEK293 cell extracts were incubated with rabbit antisera directed against SOX10 and KLF13 or anti-myc-tag monoclonals coupled to protein A sepharose beads (G&E Healthcare). For GST pulldown assays, HEK293 cell extracts were incubated with bacterially expressed and purified GST or GST-SOX10 fusion proteins bound to glutathione sepharose beads (23). After precipitation of bead-bound material by centrifugation and extensive washing of the precipitate, proteins were eluted by boiling in Laemmli sample buffer.

For Western blots, whole cell extracts or eluted precipitates were separated on 10–15% polyacrylamide-SDS gels and blotted onto nitrocellulose membranes for antibody-mediated detection of specific proteins. The following antibodies and detection reagents were used: rabbit anti-KLF9 antiserum (home-made, 1:10 000 dilution), rabbit anti-KLF13 antiserum (home-made, 1:10 000 dilution), rabbit anti-GAPDH antiserum (Santa Cruz, sc-25778,1:3000 dilution), rabbit anti-SOX10 antiserum (home-made, 1:3000 dilution), rabbit anti-MBP antiserum (Neo-markers, #RB-1460-A0, Lot# 1460A609D), guinea pig MYRF (home-made, 1:3000 dilution), mouse anti-myc-tag monoclonal (9B11, Cell Signaling, #2276, #Lot24) and horseradish peroxidase-labeled protein A (Zymed Laboratories, 1:3000 dilution). Detection was by chemiluminescence using luminol (Sigma).

### Chromatin immunoprecipitation

Primary oligodendroglial cells were treated after maintenance in proliferating conditions or after 6 days of differentiation with 1% paraformaldehyde at room temperature, lysed and used to prepare cross-linked chromatin that was subsequently sheared with a Bioruptor (Diagenode) to fragments of 150–400 bp. Sheared chromatin was precleared and incubated with rabbit anti-KLF9 antiserum, rabbit anti-KLF13 antiserum or rabbit pre-immune serum. After precipitation with BSA-blocked protein A sepharose beads, crosslink reversal at 65°C and proteinase K digestion, DNA was purified with the NucleoSpin Gel and PCR cleanup Kit (Macherey-Nagel) and used for quantitative PCR using the following primer pairs: 5′-ATCTGAATCCATGATTGTCC-3′ and 5′-GTCAGAAGTAGCCAGAGAGACTG-3′ for the *Gjc2* promoter, 5′-CCTTCTCACCAAGGGAAGGTC-3′ and 5′-GTAACCTTGGTCCCAAGAGACAG-3′ for the *Mag* promoter, 5′-CTGGTACAGTGAGCAACTCAGG-3′ and 5′-AAAACGCACAGATATGCGCAGG-3′ for the *Mbp* promoter, 5′-CAGCGTGGGAGGAGAATTCA-3′ and 5′-CCTTTGTTGGGGGCTGCT-3′ for the *Aatk* promoter, 5′- CCAGGGCATGGGAACGAA-3′ and 5′- CTCCAAACCCTCCAAACAAGC-3′ for the *Plp*1 enhancer and 5′-GTGGCTGCCAAACTAAGGTG-3′ and 5′-CCTGCTGACTGGGCCACATC-3′ or 5′- TCCAGATGTGAGAGAAAAACAA-3′ and 5′-CACCGAGTACAGAAAAGGTCCA-3′ for neighboring control regions. All samples were processed as technical triplicates. The ΔΔCt method was used to calculate the enrichment obtained in the precipitate with rabbit anti-KLF9 or anti-KLF13 antiserum over pre-immune serum.

### Mice

Generation and genotyping of KLF13-deficient and *Sox10^fl/fl^* mice have been described (24,25). Mice were kept under standard housing conditions with continuous access to food and water and 12:12 h light–dark cycles in accordance with animal welfare laws as approved by the responsible local committees and government bodies. Spinal cord tissue was obtained at postnatal days (P) 0, 7, 21 and at 2 months from both male and female animals and snap frozen for later preparation of total RNA and whole cell extracts. Alternatively tissue was processed for cryotome sectioning by fixation in 4% paraformaldehyde, dehydration in 30% sucrose and freezing in Tissue Freezing Medium (Leica) (18).

### Immunochemistry and *in situ* hybridization

Immunohistochemistry and *in situ* hybridization were performed on 10 μm transverse spinal cord cryotome sections (forelimb level). Sectioning was followed by in situ hybridization with DIG-labeled antisense riboprobes specific for *Mbp* and *Plp1* (18) or immunohistochemistry using the following primary antibodies: rat anti-MBP monoclonal (Bio-Rad, #MCA409S, Lot #210610, 1:500 dilution), mouse anti-O4 monoclonal (R&D Systems, #MAB1326, Lot#HWW1115081, 1:500 dilution), goat anti-SOX10 antiserum (home-made, 1:1000 dilution) (26), guinea pig anti-KLF9 antiserum (1:3000, home-made, this study), chicken anti-KLF13 antibodies (1:3000, home-made, this study) rabbit anti-MYRF antiserum (home-made, 1:400 dilution) (18), rabbit anti-OLIG2 antiserum (Millipore, #AB9610, Lot # 2060464, 1:1000 dilution), goat anti-PDGFRA antiserum (R&D Systems, #AF1062, Lot# E-1210, 1:50 dilution), and chicken anti-GFP antibodies (1:10 000, Aves Labs, #GFP‐1020, Lot#GFP879484). Secondary antibodies were coupled to Cy3 (Dianova, 1:200 dilution), Cy5 (Dianova, 1:200 dilution) or Alexa Fluor 488 (Molecular Probes, 1:500 dilution) fluorescent dyes. Nuclei were counterstained with 4′,6-diamidino-2-phenylindole dihydrochloride (DAPI). Some of the antibodies were also used for immunocytochemistry on cells cultured on cover slips and treated with 4% paraformaldehyde for 15 minutes. Stainings were documented with a Leica DMI6000 B inverted microscope (Leica) equipped with a DFC 360FX camera (Leica).

### Quantitative RT-PCR (qRT-PCR)

RNA was prepared from primary rodent oligodendroglial cultures under proliferative and differentiation conditions and from mouse spinal cord at P0, P7, P21 and 2 months using Trizol (Invitrogen). After reverse transcription RNA was used for quantitative PCR on a Biorad CFX96 Real Time PCR System using PowerUp SYBR Green Mastermix (Thermo Fisher Scientific). Primer pairs for *Sox10*, *Pdgfra*, *Mbp, Mag, Plp1, Acss2, Mboat1* and *Lss* have been described before (26–28). They were newly designed for *Klf9* (for rat 5′-GTCACGACCAGAGTGCTTCA-3′ and 5′-GGTGTGCTCTTGCTTGCATA-3′, for mouse 5′-AGTGCATACAGGTGAACGGC-3′ and 5′-GGGCTGGCAAGAGCCTTTTT-3′), *Klf13* (for rat 5′-GAGAAGCGCTTCATGC-3′ and 5′-GCTTTCCTATTACCAAAGGG-3′, for mouse 5′-CGAGAAAGTTTACGGGAAAT-3′ and 5′-CAGCTGAACTTCTTCTCG-3′), *Klf14* (for rat 5′- TCCTTCAAGCACAACCCCC -3′ and 5′- GCAAGACTTCCCACTACCCTCT-3′) and *Klf16* (for rat 5′-CTTCGCACCTCAAGTCACACC-3′ and 5′-GGCGTGCTTGGTCAGGT-3′). All samples were processed as technical triplicates. Transcript levels were normalized to *Rpl8*. The ΔΔCt method was used for data analysis.

### RNA-seq and analysis

Total RNA was isolated from cultured primary mouse oligodendroglia of Klf13-knockout pups and wildtype controls after 6 days of differentiation using the RNeasy Micro Kit (Qiagen). RNA samples (three Klf13-knockout and four control samples) were treated with DNaseI to remove contaminating DNA and quantified using a Qubit 4.0 fluorometer (Life Technologies). RNA integrity was checked on an Agilent 5300 Fragment Analyzer (Agilent Technologies). RNA sequencing libraries were prepared using the NEBNext Ultra II RNA Library Prep Kit for Illumina following the manufacturer's instructions (NEB), multiplexed and sequenced on an Illumina NovaSeq 6000 instrument using a 2 × 150 Pair-End (PE) configuration. On average 67 million reads were generated per library (GENEWIZ Germany GmbH, Leipzig). Sequence analysis and base calling were conducted by NovaSeq Control Software v1.7. Generated raw sequence data were converted into fastq files and de-multiplexed using Illumina bcl2fastq program version 2.20 with one mismatch allowed for index sequence identification. Sequence reads were trimmed using Trimmomatic v.0.36 and mapped to the mouse reference genome available on ENSEMBL using STAR aligner v.2.5.2b. Unique gene hit counts within exon regions were calculated using the Subread package v.1.5.2. Subsequently, DESeq2 was used for downstream differential expression analysis. Gene expression values are deposited in GEO under accession number GSE212736. To identify changes in gene expression upon loss of KLF13, a list of genes with significantly changed expression (DEGs, absolute log_2_-fold change ≥±1.0 and adjusted *P*-value of ≤0.05) was generated using the Wald test. A bi-clustering heatmap was created using the pheatmap R package by plotting log_2_ transformed expression values in samples to visualize the expression profile of the top 30 differentially expressed genes sorted by their adjusted *P*-value. Gene ontology (GO) analysis of DEGs with base mean counts ≥20 and an absolute log_2_-fold change ≥±1.5 was performed using the Gene Ontology enrichment, analysis and visualization tool GOrilla (http://cbl-gorilla.cs.technion.ac.il/) in combination with semantic clustering by REViGO (http://revigo.irb.hr/) (33). The Gene Set Enrichment Analysis (GSEA) tool from the Broad Institute (http://software.broadinstitute.org/gsea/index.jsp) was employed to determine whether a defined set of genes shows statistically significant, concordant differences between RNA samples from control and KLF13-deficient samples.

### Bioinformatic analysis of transcription factor binding

For motif discovery within DNA sequences, the DNA-pattern program of the Regulatory Sequence Analysis Tools (http://rsat.sb-roscoff.fr/) was used. Binding motifs for Klf proteins (5′-(A/C)C(G/A/T)CCC-3′) and Sox10 (5′-(A/T)CAA(A/T)-3′) were not only mapped in the oligodendrocyte-specific regulatory sequences studied in luciferase assays (see Figure 4A), but also near Sox10 ChIP-seq peaks (29) associated with genes specifically expressed in newly formed oligodendrocytes or myelinating oligodendrocytes (30). For the latter, each of the 3042 CNS-specific Sox10 peaks acc. to GSE64703 was enlarged by 250 bp on both sides and used in the bedtools program Intersect intervals (Galaxy version 2.30.0) to identify 1084 genes in the rn5 genome that have Sox10 peaks in their proximity. The Bioinformatics and Evolutionary Genomics webtools platform (http://bioinformatics.psb.ugent.be/) was used to determine a group of 282 among the 1084 genes that were expressed in newly formed or myelinating oligodendrocytes with a FPKM value ≥20. Within this group, 105 genes were solely expressed in newly formed oligodendrocytes, 170 genes in newly formed and myelinating oligodendrocytes and 7 in myelinating oligodendrocytes only. Intersect intervals was then used to map all CNS-specific Sox10 peaks in regions encompassing the 105 genes from newly formed oligodendrocytes and the 177 from myelinating oligodendrocytes genes ±10 kb up- and downstream, leading to the identification of 186 Sox10 peaks for newly formed and 257 Sox10 peaks for myelinating oligodendrocytes. The exact sequences of the Sox10 peaks (with 250 bp on each side) were then retrieved with the help of the program fetch-sequences from UCSC on the Regulatory Sequence Analysis Tools platform, and subsequently analysed for Klf binding motifs.

### Statistical analysis

To determine whether differences in transcript levels, luciferase activities, protein amounts, precipitates or cell numbers were statistically significant, a two-tailed Student's *t* test or one way Anova with Bonferroni correction was performed as indicated in the respective figure legend (*P ≤ 0.05; **P ≤ 0.01, ***P ≤ 0.001). Results from independent animals or separate experiments were treated as biological replicates (*n* ≥ 3).

## RESULTS

### Occurrence of BTEB-like Klf proteins in oligodendroglial cells

**Figure 1.**
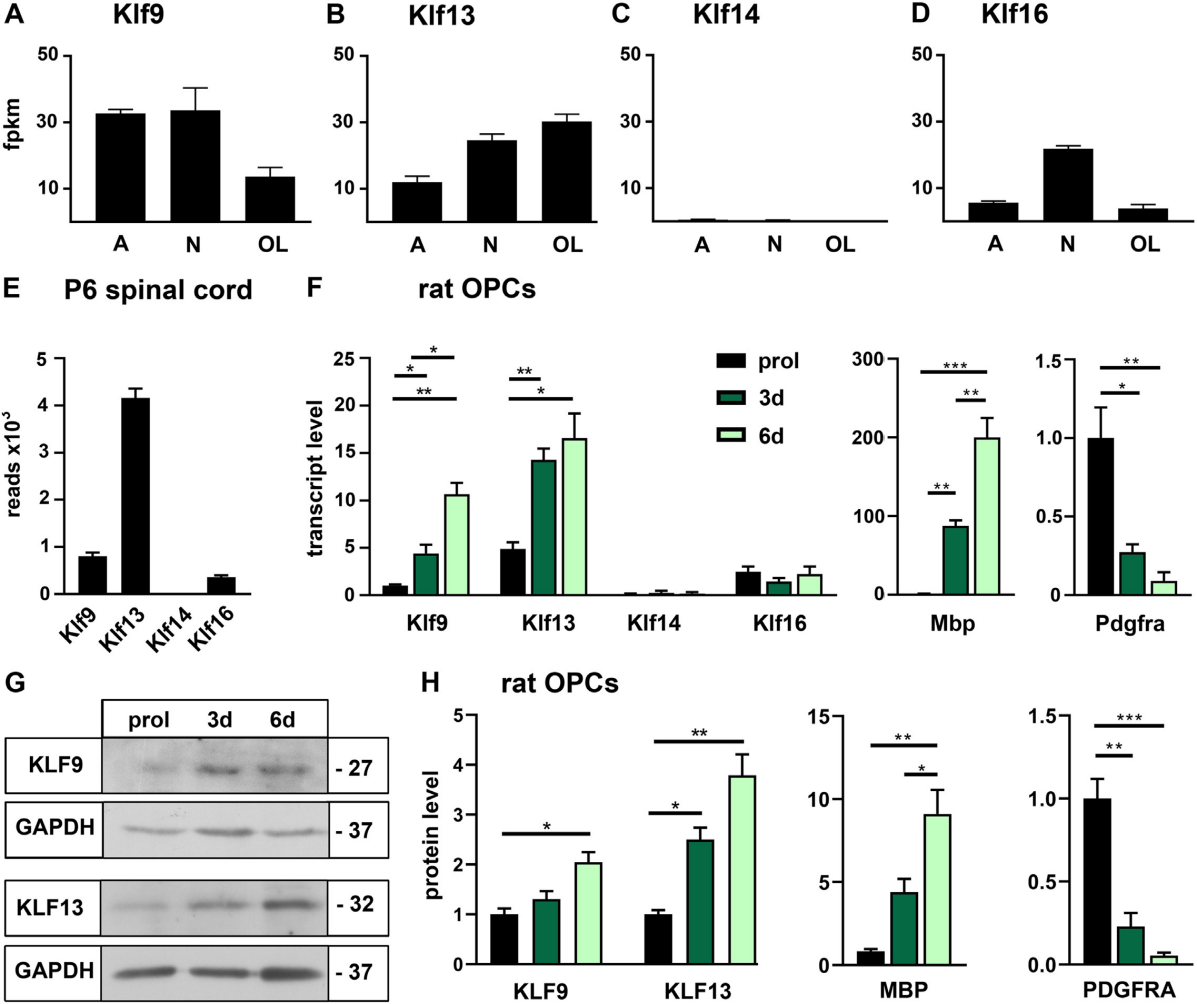
Expression of BTEB-like Klf factors in oligodendroglial cells. (**A–D**) Transcript levels for *Klf9, Klf13, Klf14* and *Klf16* in neuroectodermal cell types (A, astrocyte; N, neuron; OL, newly formed oligodendrocyte) after immunopanning at the time of birth (expressed as FPKM) according to GSE 52564 (30). (**E**) Transcript levels for *Klf9, Klf13, Klf14* and *Klf16* in oligodendroglial cells from spinal cord at P6 (expressed as read counts) according to GSE119127 (27). (**F**) Relative transcript levels for *Klf9, Klf13, Klf14*, *Klf16, Mbp and Pdgfra* in primary rat oligodendroglial cultures kept under proliferative conditions (prol) or undergoing differentiation for 3 or 6 days (3d, 6d) as determined by qRT-PCR. Normalized transcript levels for *Klf9, Mbp* and *Pdgfra* under proliferating conditions were set to 1, and levels in differentiating oligodendrocytes expressed relative to it (*n* = 3). (**G**, **H**) Protein amounts of KLF9, KLF13, MBP and PDGFRA in whole cell extracts from rat oligodendroglial cells kept under proliferating or differentiating conditions as determined by Western blot (G). Numbers on the right of the panels represent approximate size in kDa. For quantification (H), band intensities in proliferating cells were set to 1 after normalization to GAPDH levels, and values for differentiating cells expressed relative to it (*n* = 3). Statistical significance was determined by two-tailed Student's t test (F) and one way Anova (H) (*P ≤ 0.05; **P ≤ 0.01; ***P ≤ 0.001).

**Figure 2.**
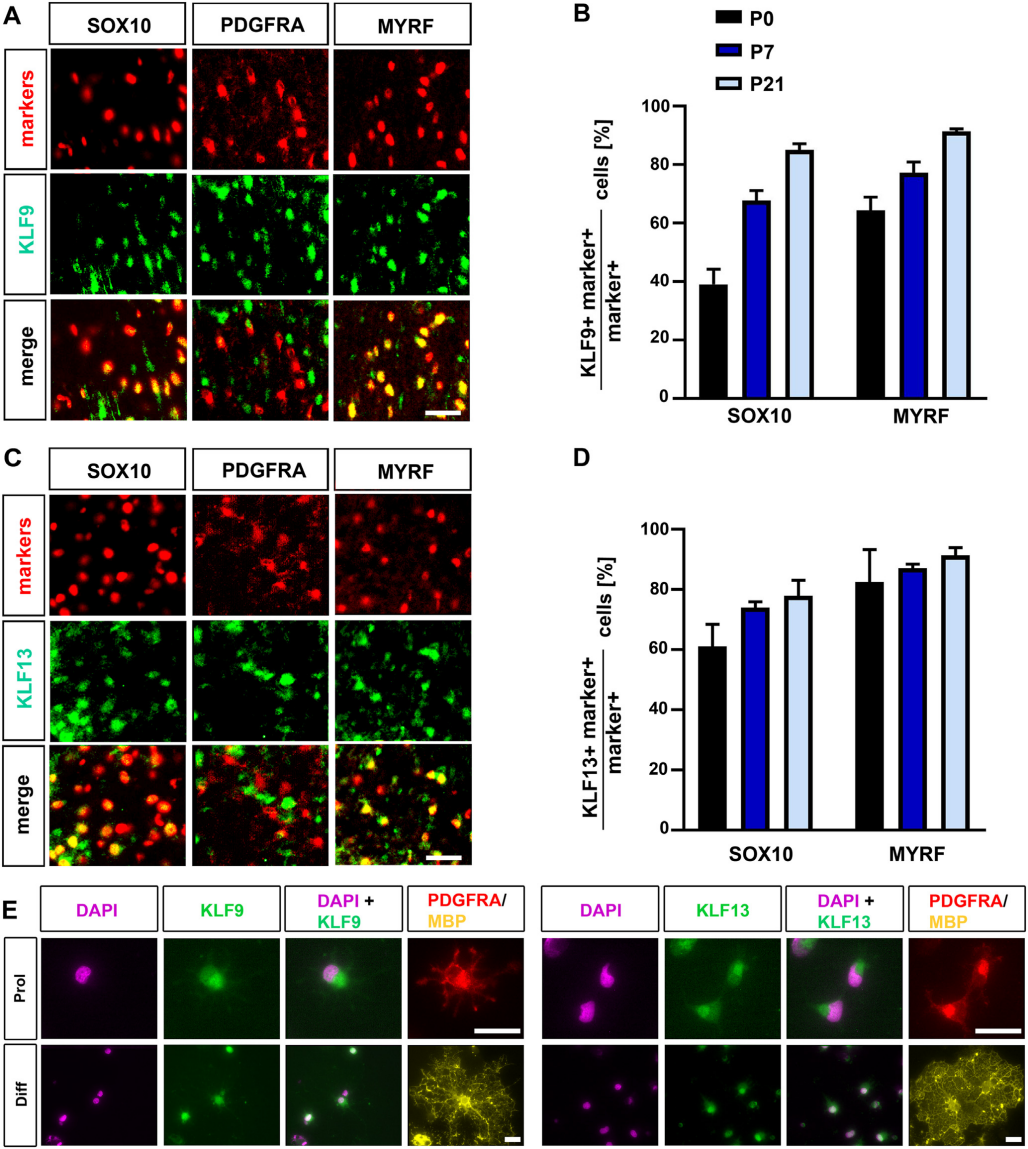
Occurrence and localization of KLF9 and KLF13 during oligodendroglial development. (**A–D**) Detection and quantification of KLF9 (A, B) and KLF13 (C, D) in oligodendroglial cells at P0, P7 and P21 following co-immunohistochemistry of Klf-specific antibodies (in green) with antibodies against SOX10, PDGFRA and MYRF (all in red). Exemplary pictures (A, C) were taken from the ventrolateral white matter of spinal cord tissue at P7. Quantification of the fraction of SOX10-positive oligodendroglial cells and MYRF-positive oligodendrocytes in the white matter that co-expressed KLF9 (B) or KLF13 (D). (**E**) Subcellular localization of KLF9 and KLF13 proteins by microscopy following co-immunohistochemistry of KLF-specific antibodies (in green) with antibodies against PDGFRA (red) and MBP (yellow), combined with a DAPI nuclear counterstain (magenta) in primary oligodendroglial cells cultured under proliferating (upper row) or differentiating (lower row) conditions. Scale bar: 50 μm (A, C), 25 μm (E).

To better understand the role of KLF9 in oligodendroglial development, we first compared its expression to other Klf proteins in these cells. We restricted our analysis to the close relatives within the BTEB subgroup of Klf proteins. Available expression data indicated substantial expression of *Klf9* and *Klf13* (30). However, according to these data neither *Klf9* nor *Klf13* transcripts were restricted to oligodendroglial cells, but were also present at high levels in neurons and astrocytes (Figure 1A, B). Compared to *Klf9* and *Klf13*, transcripts for *Klf16* occurred at much lower levels in oligodendroglial cells and *Klf14* expression was virtually undetectable in any of the major neuroectodermal cell types of the CNS (Figure 1C, D). RNA-seq data from oligodendroglial cells isolated from spinal cord at P6 and purified by FACS (27) confirmed the prominent occurrence of *Klf9* and *Klf13* in oligodendroglial cells and indicated that *Klf13* exhibits an even stronger expression during the active phase of myelination than *Klf9* (Figure 1E). In cultured oligodendroglial cells, both Klf9 and Klf13 amounts increased substantially during differentiation both on transcript level as determined by quantitative RT-PCR (qRT-PCR) (Figure 1F) and on protein level as determined by Western blot (Figure 1G, H) in a manner qualitatively similar to Mbp and reciprocal to Pdgfra levels. In contrast, *Klf14* and *Klf16* levels remained unaffected by a change from proliferation to differentiation promoting conditions (Figure 1F). By immunohistochemistry on postnatal spinal cord, KLF9 and KLF13 proteins were detectable from P0 onwards in a substantial fraction of SOX10- and MYRF-positive cells, but were absent from the majority of PDGFRA-positive OPCs at all times analysed (Figure 2A–D). The fraction of co-labelled SOX10-positive cells increased from 39 ± 9% at the day of birth for KLF9 and 61 ± 8% for KLF13 to 67 ± 6% for KLF9 and 74 ± 2% for KLF13 during the first week. For MYRF-positive cells, the increase was from 65 ± 8% for KLF9 and 76 ± 18% for KLF13 to 77 ± 7% for KLF9 and 87 ± 1% for KLF13 (Figure 2B,D). The first postnatal week coincides with the active phase of oligodendroglial differentiation and myelination. At P21, the fraction of MYRF-positive cells that simultaneously expressed KLF9 or KLF13 amounted to 92 ± 1% for KLF9 and 91 ± 3% for KLF13.

When subcellular localization of KLF9 and KLF13 was compared in oligodendroglial cultures between the few PDGFRA-positive OPCs that expressed low amounts of the Klf proteins and MBP-positive oligodendrocytes, an increased nuclear presence was observed for both Klf proteins in differentiating cells (Figure 2E). In summary, our data argue that KLF9 and KLF13 are the predominant BTEB-like Klf proteins in oligodendroglial cells and that changes in their expression level and subcellular localization are linked to the differentiation process.

### Influence of thyroid hormone and SOX10 as upstream regulators of KLF9 and KLF13

**Figure 3.**
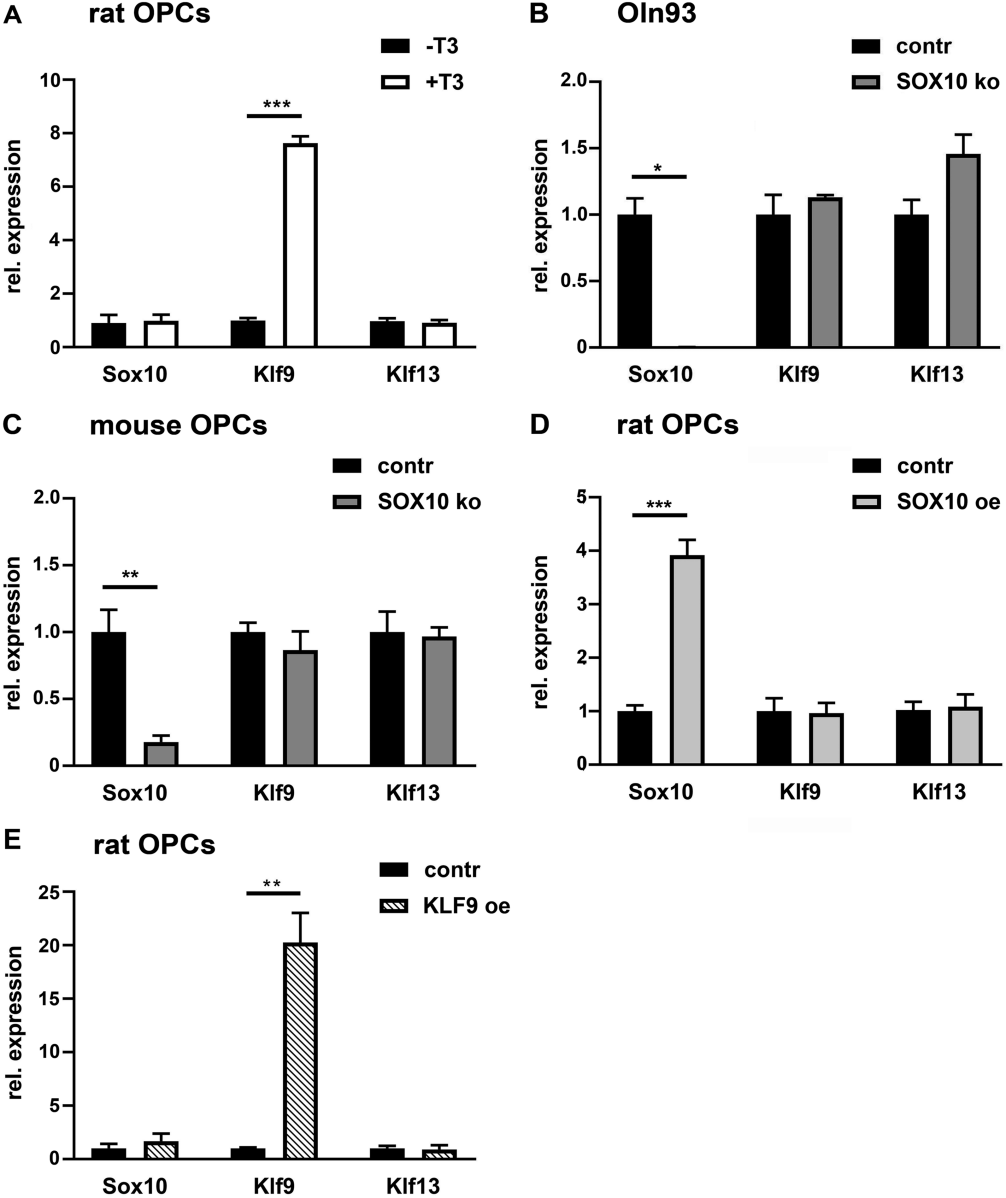
Influence of T3 and SOX10 on oligodendroglial Klf9 and Klf13 expression. (**A**) Determination of *Sox10*, *Klf9* and *Klf13* transcript levels in primary rat oligodendroglial cultures kept under differentiating conditions in the absence (–T3) or presence (+T3) of thyroid hormone by qRT-PCR. Expression levels for each gene in the absence of T3 were set to 1 ± SEM (*n* = 3). (**B**) Relative expression of *Sox10*, *Klf9* and *Klf13* in the Oln93 cell line (contr) as compared to genome-edited clonal derivatives with *Sox10* gene inactivation (SOX10 ko) according to GSE136659 (13). Expression levels for each gene in control Oln93 cells were set to 1 ± SEM (*n* = 3). (**C**) qRT-PCR determining *Sox10*, *Klf9* and *Klf13* expression in primary oligodendroglial cells from *Sox10^fl/fl^* mice (contr) as compared to cells treated with TAT-Cre (SOX10 ko) prior to a 3-day cultivation under differentiating conditions. Expression levels for each gene in untreated cells were set to 1 ± SEM (*n* = 3). (**D, E**) qRT-PCR determining *Sox10*, *Klf9* and *Klf13* expression in rat oligodendroglial cells transduced with GFP (contr in D,E), GFP and SOX10 (SOX10 oe in D) or GFP and KLF9 (KLF9 oe in E) co-expressing retrovirus. Expression levels for each gene in GFP-transduced cells were set to 1 ± SEM (*n* = 3).

To understand how KLF9 and KLF13 integrate into the oligodendroglial gene regulatory network, we first studied their induction by thyroid hormone (T3). Previous studies had shown that *Klf9* expression was robustly induced by T3 in oligodendroglial cells even when cultured under proliferative conditions in the presence of mitogens (8,9). Our own experiments reproduced the T3-dependent induction of *Klf9* expression (Figure 3A). At the same time, we failed to detect similar alterations of *Klf13* or *Sox10* levels. It thus appears that the expression of the two closely related Klf factors differs in their T3-responsiveness.

In a second set of experiments, we analysed the impact of the central transcriptional regulator and lineage determinant SOX10 on the expression of the two Klf factors. In the rat oligodendroglial cell line Oln93, CRISPR/Cas9-mediated inactivation of the *Sox10* gene did not affect the expression levels of *Klf9* or *Klf13* (Figure 3B). Similarly, primary oligodendroglial cells from *Sox10^fl/fl^* mice exhibited comparable amounts of *Klf9* and *Klf13* transcripts independent of whether the cells were treated with TAT-Cre and had lost SOX10 expression before differentiation and RNA isolation (Figure 3C). Expression of the two Klf factors also remained unchanged in rat primary oligodendroglial cultures after SOX10 overexpression following retroviral transduction (Figure 3D). Expression of *Klf9* and *Klf13* is thus independent of SOX10 in oligodendroglial cells.

Finally, we studied the consequences of KLF9 overexpression on *Klf13* expression in primary oligodendroglial cultures. Again we failed to detect any alterations of *Klf13* (and *Sox10*) transcript levels arguing that Klf13 expression is not downstream of KLF9 in oligodendroglial cells (Figure 3E).

### Synergistic activation of SOX10-responsive regulatory regions from myelin genes

**Figure 4.**
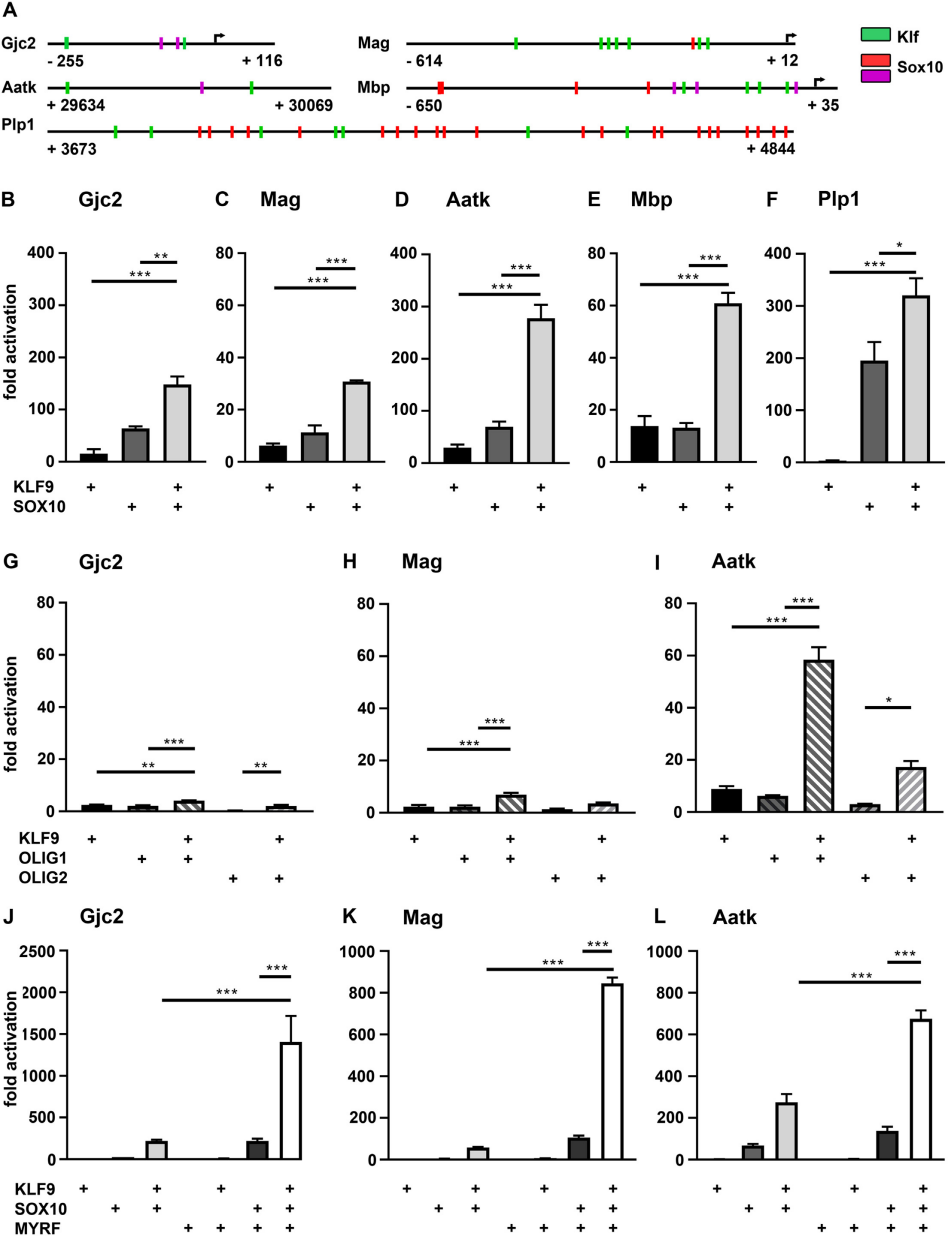
Transcriptional activity of KLF9 on regulatory regions from differentiating oligodendrocytes. (**A**) Localization of predicted Klf binding motifs (green boxes) within the oligodendroglial regulatory regions from the *Gjc2*, *Mag*, *Aatk*, *Mbp* and *Plp1* genes relative to predicted (red boxes) and validated (magenta boxes) SOX10 binding sites. For each regulatory region, first and last positions relative to the transcriptional start site (arrow) are given. (**B–L**) Luciferase assays in N2a cells transiently transfected with reporter genes under control of regulatory regions from the *Gjc2* (B, G, J), *Mag* (C, H, K), *Aatk* (D, I, L), *Mbp* (E) and *Plp1* (F) genes in the presence (+) of various combinations of KLF9 (B–L), SOX10 (B–F, J–L), OLIG1 (G–I), OLIG2 (G–I) and MYRF (J–L) as indicated below the bars. Effector-dependent activation rates are presented as fold inductions ± SEM with transfections in the absence of effectors arbitrarily set to 1 for each reporter construct (*n* = 3). Differences were statistically significant as determined by one way Anova with Bonferroni correction (*P ≤ 0.05; **P ≤ 0.01; ***P ≤ 0.001).

We have previously characterized a number of regulatory regions within or near the *Gjc2* (*Connexin 47*), *Mag*, *Aatk, Mbp* and *Plp1* genes that are active in oligodendrocytes (29) and mediate SOX10-dependent gene activation during terminal differentiation (18,20,21). All regions contained at least one and usually several predicted Klf binding motifs arguing that they may also be targeted by KLF9 and KLF13 (Figure 4A). A specific pattern in distance or orientation to known or predicted SOX10 binding sites was not detected.

For a better understanding of the molecular mechanism of Klf function, we chose to test these regulatory regions in transiently transfected N2a cells for their response towards Klf proteins in luciferase reporter assays. We started out with KLF9 and compared the effect of its presence with the well known activation by SOX10 (Figure 4B–F). KLF9 activated all regulatory regions between 4 ± 1-fold for the intronic *Plp1* and 29 ± 6-fold for the intronic *Aatk* enhancer (Figure 4B–F). Activation rates were, however, fairly modest in general and with the exception of the *Mbp* promoter below the rates obtained with SOX10 for the corresponding reporter constructs. Therefore, it was intriguing to see that all luciferase reporters exhibited a high activation in the joint presence of KLF9 and SOX10 (Figure 4B–F). Activation rates were in all cases more than additive and most impressive for the *Aatk* enhancer where joint activation rates were ∼3-fold higher than the sum of single activation rates (Figure 4D).

As most of these regulatory regions additionally bind Olig proteins according to ChIP-seq experiments (31), we also used them to study functional interactions between KLF9 and OLIG1 or OLIG2. These co-transfections, however, provided little indication for substantial cooperative effects between KLF9 and the Olig proteins except for the *Aatk* enhancer (Figure 4G-I). On this regulatory region, KLF9 was much more effective in combination with OLIG1 than with OLIG2 (Figure 4I). This is in line with previous reports that ascribe a more prominent role during oligodendrocyte differentiation to OLIG1 than OLIG2 (32).

As the third major driver of myelin gene expression in differentiating oligodendrocytes we then tested the MYRF transcription factor (12) for its ability to functionally cooperate with KLF9. The combination of KLF9 and MYRF was not more active on any of the analysed regulatory regions than KLF9 alone (Figure 4J–L). We had previously shown, however, that MYRF and SOX10 strongly activated these regions together (13,18). Therefore, we also performed reporter assays in N2a cells co-transfected with KLF9, MYRF and SOX10. This particular combination of transcription factors yielded activation rates for each of the regulatory regions that were substantially higher than obtained in parallel transfections for either KLF9 and SOX10 or SOX10 and MYRF (Figure 4J–L). The magnitude of the obtained cooperative effect was impressive for all analysed regulatory regions. The *Gjc2* promoter, for instance, experienced a ca. 220-fold activation in the presence of KLF9 and SOX10 as well as in the presence of SOX10 and MYRF. The triple combination of KLF9, SOX10 and MYRF elicited a massive 1408-fold activation (Figure 4J). We conclude from these transfections that KLF9 likely cooperates with SOX10 in the activation of oligodendroglial differentiation and myelin genes and that MYRF is a further reinforcing partner in this induction event.

**Figure 5.**
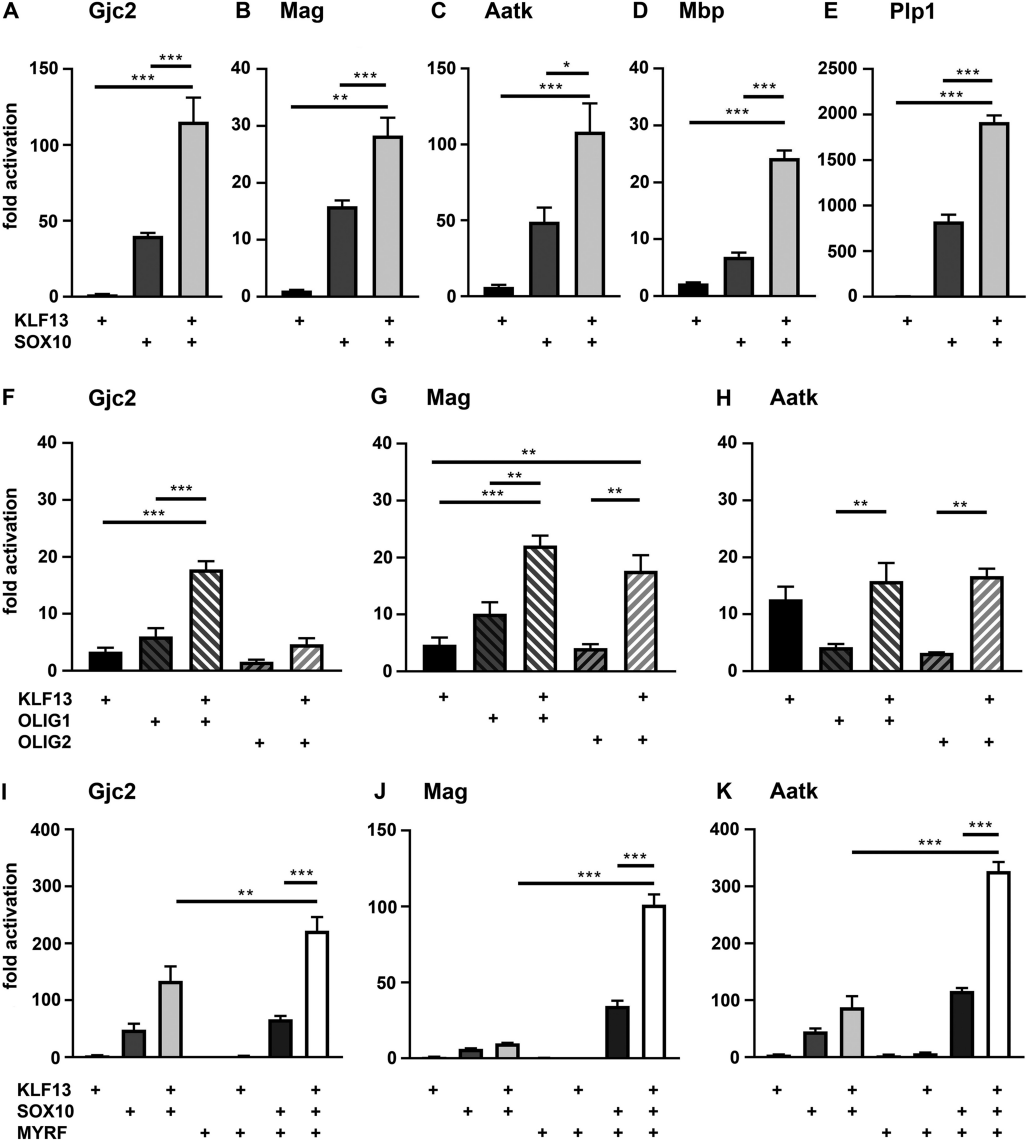
Transcriptional activity of KLF13 on regulatory regions from differentiating oligodendrocytes. (**A–K**) Luciferase assays in N2a cells transiently transfected with reporter genes under control of regulatory regions from the *Gjc2* (A, F, I), *Mag* (B, G, J), *Aatk* (C, H, K), *Mbp* (D) and *Plp1* (E) genes in the presence (+) of various combinations of KLF13 (A–K), SOX10 (A–E, I–K), OLIG1 (F–H), OLIG2 (F–H) and MYRF (I–K) as indicated below the bars. Effector-dependent activation rates are presented as fold inductions ± SEM with transfections in the absence of effectors arbitrarily set to 1 for each reporter construct (*n* = 3). Differences were statistically significant as determined by one way Anova with Bonferroni correction (*P ≤ 0.05; **P ≤ 0.01; ***P ≤ 0.001).

For comparison, we also analysed KLF13 for its effect on the various regulatory regions (Figure 5A–E). Despite differences in the exact fold inductions, KLF13 exhibited activation rates that were overall comparable to KLF9. In combination with SOX10, we again observed more than additive activation rates for all regulatory regions tested. Differences between KLF9 and KLF13 were a bit more pronounced in their respective cooperation with Olig proteins (Figure 5F–H). KLF13 was more broadly active in combination with Olig proteins and less discriminatory in its choice between OLIG1 and OLIG2 than KLF9. However, joint activation rates remained below levels obtained with SOX10.

Importantly, KLF13 also displayed transcriptional activity in combination with SOX10 and MYRF on all analysed regulatory regions, although the obtained superstimulation rates were not as impressive as those for KLF9 (Figure 5I–K). Despite these quantitative differences, KLF9 and KLF13 function in a qualitatively similar manner in reporter gene assays.

**Figure 6.**
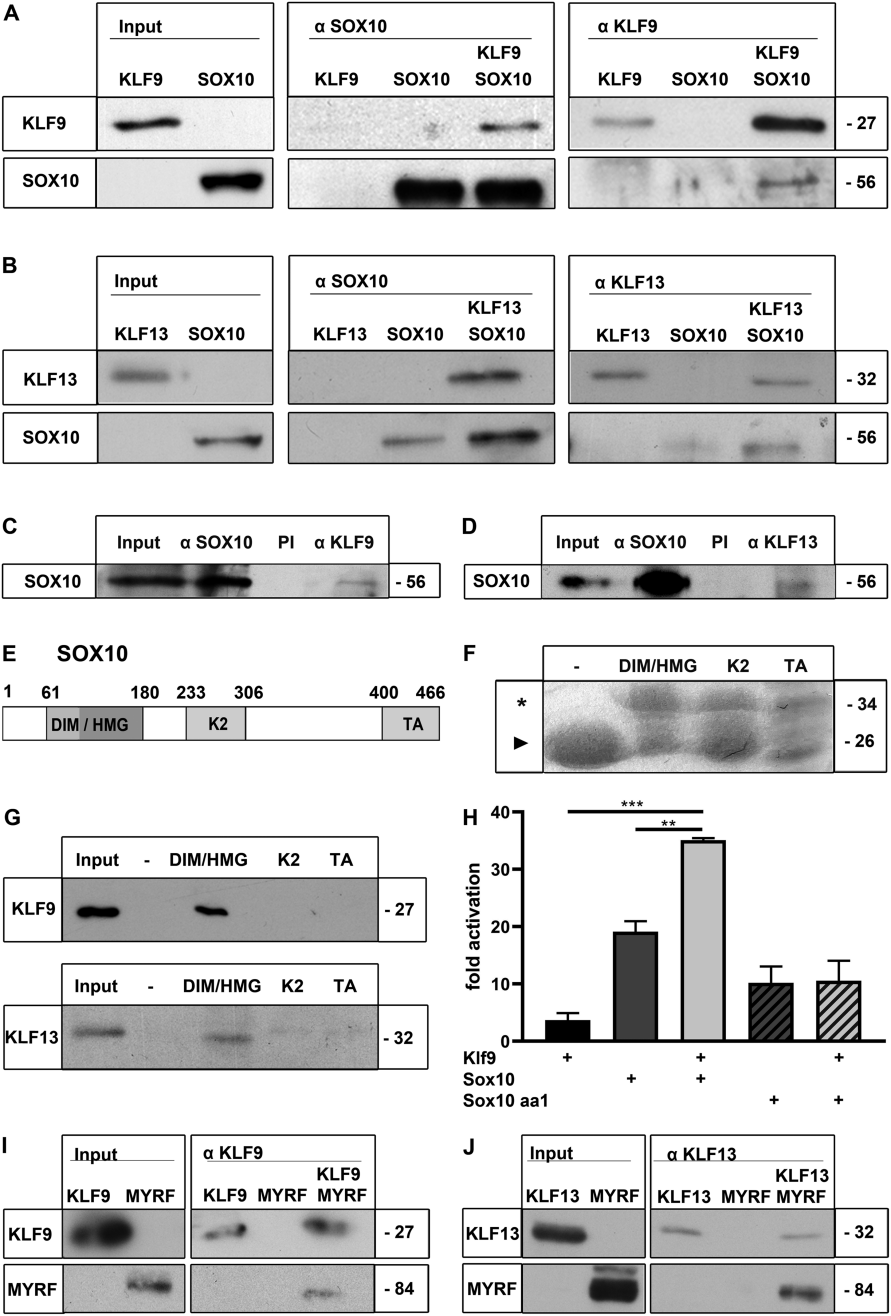
Physical interaction of Klf proteins with SOX10 and MYRF. (**A**, **B**) Co-immunoprecipitation of KLF9 (A) or KLF13 (B) with SOX10 from extracts of transfected HEK293 cells. The Western blot on the left shows the input. The middle panel is from a representative experiment in which HEK293 extracts containing either the Klf protein, SOX10 or a combination of both underwent precipitation after incubation with anti-SOX10 (αSOX10) antibodies. The right panel shows the results from precipitation with anti-myc (αKLF9) or anti-KLF13 (αKLF13) antibodies. (**C**, **D**) Immunoprecipitation of SOX10 from extracts of primary oligodendroglial cells kept for at least 3 days under differentiating conditions using antisera directed against SOX10 (αSOX10), KLF9 (αKLF9, C) or KLF13 (αKLF13, D) as well as the respective pre-immune sera (PI). The Western blots show SOX10 input and precipitates. (**E**) Domain structure of Sox10: DIM/HMG, dimerization and HMG-domain; K2, central protein-protein interaction and transactivation domain; TA, carboxy-terminal transactivation domain. (**F**) Coomassie stain of polyacrylamide-SDS-gel showing GST (–, marked by arrowhead) and fusions between GST and functional SOX10 domains (23) (marked by asterisk) after expression in bacteria, purification and binding to glutathione sepharose beads. (**G**) Bead-bound KLF9 (upper blot) and KLF13 (lower blot) were visualized after GST pulldown from HEK293 extracts (input) by Western blot with anti-myc antibodies for KLF9 or anti-KLF13 antibodies. (**H**) Luciferase assays in N2a cells transiently transfected with the *Gjc2* luciferase reporter in the presence (+) of various combinations of KLF9, SOX10 and the SOX10 aa1 mutant as indicated below the bars. Effector-dependent activation rates are presented as fold inductions ± SEM with transfections in the absence of effectors arbitrarily set to 1 (*n* = 3). Differences were statistically significant as determined by one way Anova with Bonferroni correction (*P ≤ 0.05; **P ≤ 0.01; ***P ≤ 0.001). (**I, J**) Co-immunoprecipitation of MYRF with KLF9 (I) or KLF13 (J) from extracts of transfected HEK293 cells containing either the Klf protein, MYRF or a combination of both. The Western blot on the left shows the input. The blot on the right shows the results from precipitation with antisera against KLF9 (αKLF9) or KLF13 (αKLF13). Numbers on the right side of blots indicate the approximate molecular weight of proteins in kDa.

For detailed insight into the functional interaction with SOX10, we checked for the ability of Klf proteins to physically interact with SOX10. Co-immunoprecipitation experiments on extracts from HEK293 cells transfected with myc-tagged KLF9, SOX10, or both resulted in KLF9 precipitation with antibodies against SOX10 when SOX10 was present in the extract (Figure 6A, middle panel). Similarly, SOX10 was precipitated from KLF9-containing HEK293 cell extracts with anti-myc-tag antibodies (Figure 6A, right panel). When KLF9 was replaced by KLF13 in the co-immunoprecipitation experiments, similar results were obtained (Figure 6B). Interactions between SOX10 and the two Klf proteins were also detected in extracts from primary oligodendroglial cells kept for 3 days or more under differentiating conditions in cell culture by co-immunoprecipitation of SOX10 with antibodies directed against KLF9 or KLF13 (Figure 6C, D).

To further confirm the interaction, GST pulldown experiments were carried out with conserved domains of the SOX10 protein (Figure 6E). The domains were produced as recombinant GST fusion proteins in bacteria, bound in sufficient amounts to glutathione-sepharose beads (Figure 6F) and incubated with KLF9- or KLF13-containing HEK293 extracts. By pulldown, the physical interaction of both Klf proteins was mapped to the SOX10 region encompassing dimerization and adjacent HMG domains (Figure 6G). Reporter assays with luciferase under control of the *Gjc2* promoter provided additional evidence for the relevance of the dimerization domain in the context of synergistic gene activation. As the essential SOX10 binding sites in the *Gjc2* promoter are monomeric (21), it can still be activated in N2a cells by the dimerization-deficient aa1 mutant (position 71–73 CIR to AAA) of SOX10 (33). However, Klf proteins were only able to cooperate with wildtype SOX10 and not with the aa1 mutant as exemplified for KLF9 (Figure 6H). Both KLF9 and KLF13 also physically interacted with MYRF as evident by co-immunoprecipation of MYRF from extracts of transfected HEK293 cells using antibodies against KLF9 and KLF13 (Figure 6I,J).

### Oligodendroglial development and CNS myelination in KLF13-deficient mice

**Figure 7.**
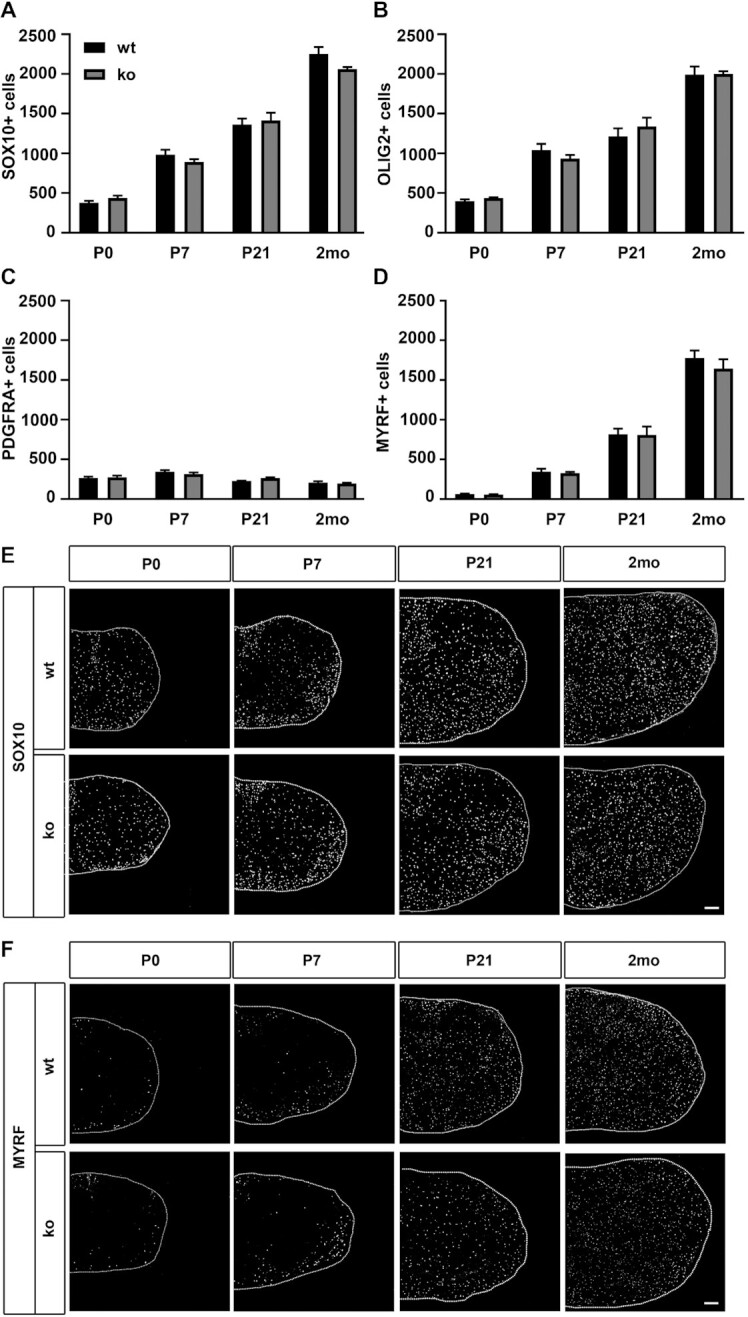
Oligodendroglial development in KLF13-deficient mice. (**A–D**) Quantification of the number of SOX10-positive (A) and OLIG2-positive (B) oligodendroglial cells, PDGFRA-positive OPCs (C) and MYRF-positive premyelinating and myelinating oligodendrocytes (D) in the spinal cord (forelimb level) of KLF13-deficient mice (ko, gray bars) and age-matched controls (wt, black bars) following immunohistochemistry with respective antibodies at P0, P7, P21 and 2 months (2mo). Three sections per animal and 3 animals per genotype were quantified. Mean numbers per section ± SEM are shown. No statistical significance was detected when ko and wt were compared at a particular time point by Student's t test. (**E, F**) Representative immunohistochemical stainings of spinal cord sections (right half) at P0, P7, P21 and 2 months for SOX10 (E) and MYRF (F). Scale bars: 100 μm.

Developmental myelination in KLF9-deficient mice was reported normal (8). Our new results point to a substantial functional overlap between KLF9 and KLF13 during this process as one likely explanation. In contrast to KLF9 deletion mutants, KLF13-deficient mice (24) were not yet analyzed with respect to oligodendroglial development. To fill this gap, we studied oligodendroglial cells in the spinal cord of KLF13-deficient mice in the early postnatal period of active myelination (P0 – P21) and during early adulthood (2 months). First we quantified the total number of oligodendroglial cells by immunohistochemistry using antibodies directed against the lineage markers SOX10 and OLIG2 (Figure 7A, B, E). At all time points analyzed, oligodendroglial cell numbers in KLF13-deficient spinal cords were comparable to those from control littermates. We were unable to detect differences in the number of PDGFRA-positive OPCs (Figure 7C) or MYRF-positive premyelinating and myelinating oligodendrocytes that substantially increase during the first two months (Figure 7D, F). Microscopic inspection of the sections furthermore revealed a normal distribution of oligodendroglial cells and differentiating oligodendrocytes, arguing that prenatal OPC migration occurs normally and that oligodendrocytes start their differentiation in the future white matter regions even in the absence of KLF13 (Figure 7E, F).

**Figure 8.**
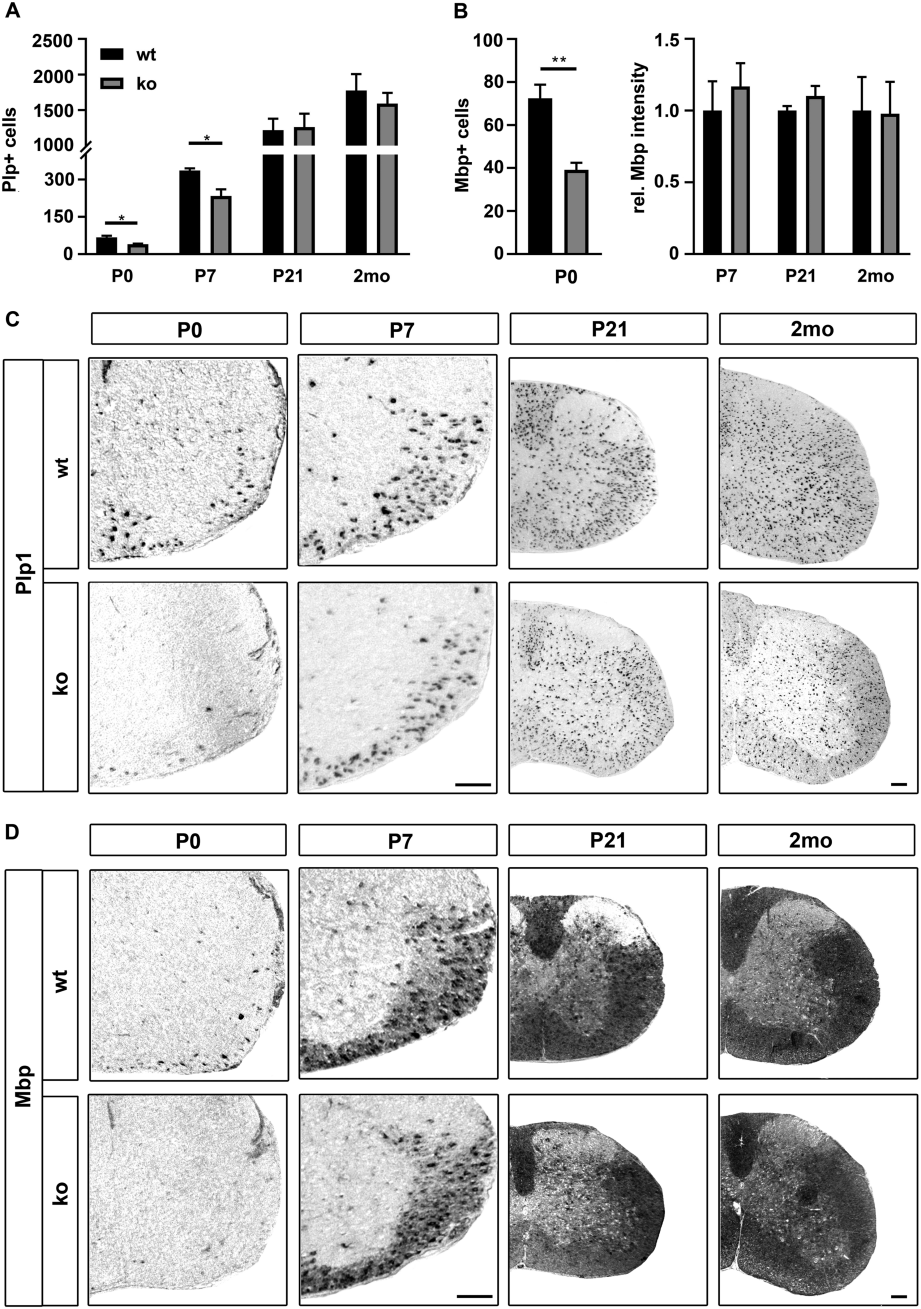
Myelin gene expression in KLF13-deficient mice. (**A–D**) Quantification of *Plp1*- (A) and *Mbp*- (B) expressing oligodendrocytes in the spinal cord (forelimb level) of KLF13-deficient mice (ko, gray bars) and age-matched controls (wt, black bars) following in situ hybridization with antisense probes directed against *Plp1* (**C**) and *Mbp* (**D**) at P0, P7, P21 and 2 months (2mo). For *Plp1* at all time points and for *Mbp* at P0, positive cells were counted on three sections per animal and three animals per genotype. Mean numbers per section ± SEM are shown. For *Mbp* at P7, P21 and 2 months (2mo) signal intensities were measured. Statistical significance between ko and wt was determined at each time point by Student's *t* test (*P ≤ 0.05). For representative *in situ* hybridizations (C, D), the ventral right half of the spinal cord is shown at P0 and P7 and the complete right half for P21 and 2mo. Scale bar: 100 μm.

**Figure 9.**
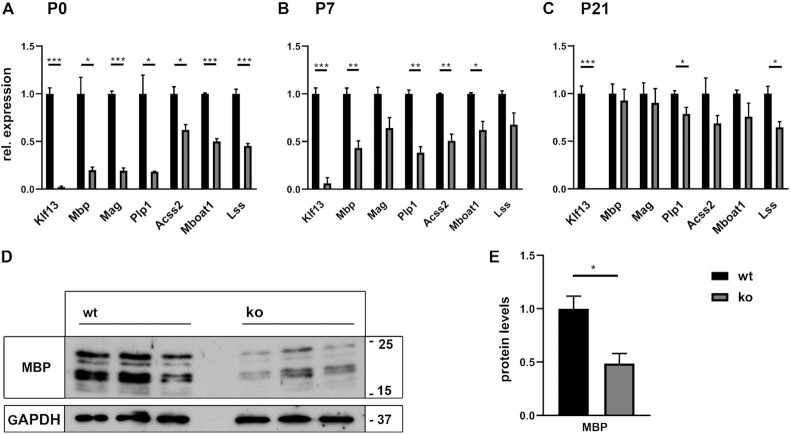
Myelination parameters in KLF13-deficient mice. (**A–C**) Quantitative RT-PCR on RNA from spinal cord tissue to determine *Klf13, Mbp, Mag, Plp1, Acss2, Mboat1* and *Lss* transcript levels in KLF13-deficient mice (ko, gray bars) and age matched controls (wt, black bars) at P0 (A), P7 (B) and P21 (C). (**D, E**) Protein amounts of MBP in whole cell extracts from spinal cord of KLF13-deficient mice and age matched controls at P3 as determined by Western blot (D). Numbers on the right of the panels represent approximate size in kDa. For quantification (E), band intensities in wt extracts were set to 1 after normalization to GAPDH levels, and normalized values for ko extracts expressed relative to it (*n* = 3). Statistical significance between ko and wt was determined by Student's *t* test (*P ≤ 0.05).

When *Plp1* and *Mbp* transcripts were analyzed as markers of the beginning myelination process by in situ hybridization, we detected a statistically significant reduction in the number of *Plp1*-positive cells at P0 and P7, and in the number of *Mbp*-expressing cells at P0 (Figure 8A–D). In situ hybridizations and quantifications at later stages failed to reveal differences to controls, arguing that oligodendrocytes in the KLF13-deficient spinal cord exhibit a mild and transient delay in myelination. In support of this conclusion, *Plp1* and *Mbp* transcript levels were also substantially reduced in RNA prepared from spinal cord of KLF13-deficient mice as compared to controls at P0 (Figure 9A). The reduction was already less pronounced at P7 and largely disappeared by P21 (Figure 9B, C). Transiently lower transcript levels were also observed in qRT-PCR studies for the myelin gene *Mag* and the lipid biosynthetic genes *Acss2, Mboat1* and *Lss* arguing that the delay is not restricted to *Plp1* and *Mbp* expression, but broadly concerns the myelination program. Additionally, the reduction of MBP was not only seen on transcript level, but also occurred on protein level. Western blots confirmed lower amounts of MBP protein in spinal cord extracts from KLF13-deficient mice at P3 (Figure 9D, E). All available data therefore point to a mild and transient myelination delay in the spinal cord of KLF13-deficient mice.

### Impact of KLF13 on gene expression in differentiating oligodendroglial cells

**Figure 10.**
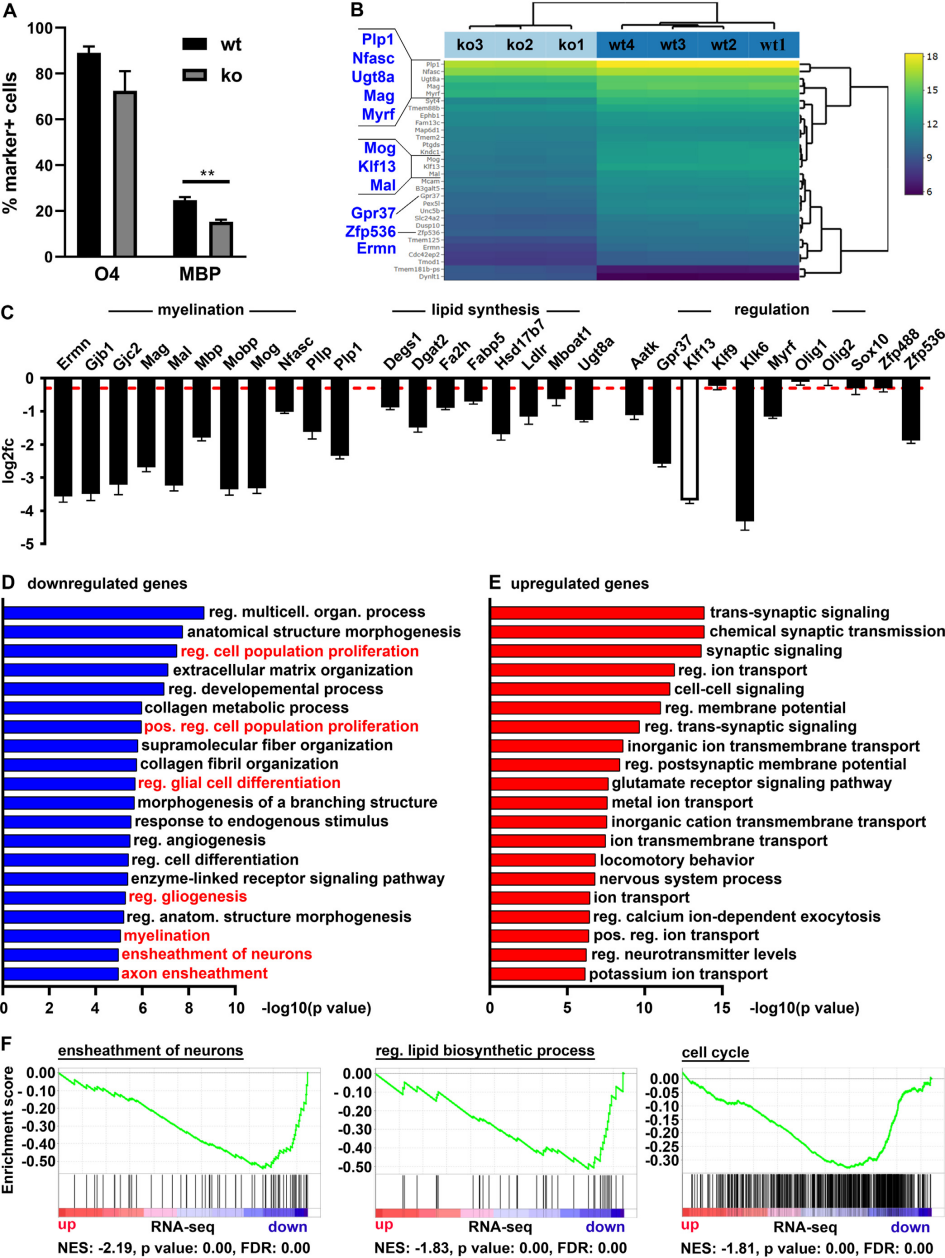
Cell-intrinsic role of KLF13 in oligodendroglial differentiation and myelin gene expression. (**A**) Quantification of the percentage (±SEM) of O4-positive and MBP-positive cells among all oligodendroglial cells from control (wt, black bars) or KLF13-deficient mice (ko, gray bars) after 6 days of culture in differentiating conditions (*n* = 3). (**B–F**) Bioinformatic analysis of RNA-seq studies performed on KLF13-deficient (ko, *n* = 3) and wildtype control (wt, *n* = 4) oligodendroglial cells cultured in differentiating conditions. (B) Profile of the top 30 differentially expressed genes sorted by their adjusted *P*-value and depicted in a bi-clustering heatmap by plotting their log_2_ transformed expression values. (C) Changed expression of genes related to myelination, lipid synthesis and regulation of oligodendroglial differentiation in KLF13-deficient oligodendrocytes. (D) GO analysis of the 278 genes down-regulated with a log_2_fold change ≥ -1.5 (*P*-value ≤ 0.05) in KLF13-deficient oligodendrocytes. (E) GO analysis of the 301 genes up-regulated with a log_2_fold change ≥ 1.5 (*P*-value ≤ 0.05) in KLF13-deficient oligodendrocytes. (F) GSEA for genes related to ensheathment of neurons, regulation of lipid biosynthetic process and cell cycle in KLF13-deficient oligodendrocytes. NES, normalized enrichment score; FDR, false discovery rate.

In our mouse model, KLF13 is constitutively deleted in all tissues and cell types. Therefore, it is formally possible that the observed delay in myelination is not a cell-intrinsic effect in the oligodendrocytes. To analyse whether KLF13 is part of the regulatory network for myelination within oligodendrocytes, we generated primary oligodendroglial cultures from KLF13-deficient mice and wildtype controls and analysed their ability to express differentiation markers. Immunocytochemical staining for the early differentiation marker O4 and the myelin gene *Mbp* revealed that significantly fewer KLF13-deficient than control oligodendroglial cells had reached an MBP-positive state after 6 days of differentiation thereby supporting a cell-intrinsic function of KLF13 (Figure 10A).

The effect of KLF13 on oligodendroglial differentiation was also confirmed on a global scale by RNA-seq studies on KLF13-deficient oligodendrocytes cultured under differentiating conditions. We found 11 myelin and oligodendroglial differentiation genes among the top 30 differentially expressed genes (DEGs) as shown in a bi-clustering heatmap with sorting by adjusted *P*-value (Figure 10B). A more thorough overview of expression changes of genes related to myelination, lipid synthesis and regulation of oligodendroglial differentiation is presented in Figure 10C. Note that neither *Klf9* nor *Sox10* or the Olig factors exhibited a significantly changed expression in KLF13-deficient cells. Gene ontology (GO) and gene set enrichment analysis (GSEA) of the 278 significantly downregulated genes (log_2_fold ≥ –1.5; *P*-value ≤ 0.05; base mean count ≥ 20) revealed an enrichment of terms associated with glial differentiation, myelination and ensheathment of neurons (Figure 10D, F). Other interesting terms for the downregulated genes pointed to substantial changes in proliferation and extracellular matrix organization that are known to coincide with oligodendrocyte differentiation. Terms referring to trans-synaptic signaling, ion transport and regulation of membrane potential in corresponding GO and GSEA analyses for the 301 significantly upregulated genes (log_2_fold ≥ 1.5; *P*-value ≤ 0.05; base mean count ≥ 20) point to persistent expression of OPC-specific ion channels in KLF13-deficient cells and are indicative of a less differentiated state (Figure 10E, F). These studies impressively confirm the impact of KLF13 on oligodendrocyte differentiation.

### Functional redundancy between KLF9 and KLF13 in oligodendrocyte differentiation

**Figure 11.**
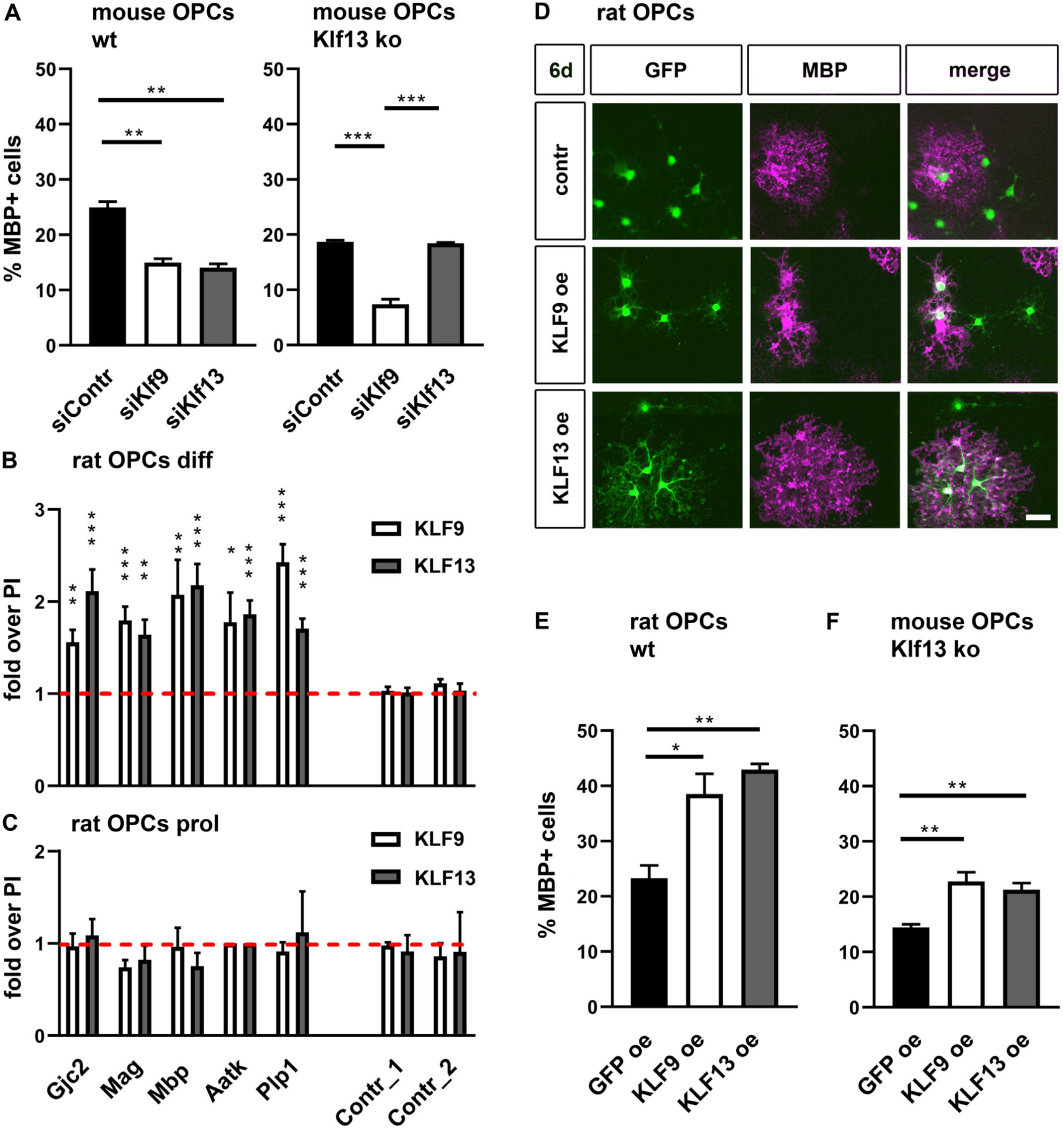
Functional redundancy of KLF9 and KLF13 in oligodendroglial differentiation and myelin gene expression. (**A**) Quantification of the percentage (± SEM) of MBP-positive cells among all oligodendroglial cells from wildtype (wt, left panel) or KLF13-deficient mice (Klf13 ko, right panel) after 6 days of culture in differentiating conditions following transfection of siRNA pools against *Klf9* (siKlf9) or *Klf13* (siKlf13) and a control siRNA pool (siContr) as indicated below the bars (*n* = 3). (**B**, **C**) Chromatin immunoprecipitation on rat oligodendroglial cells cultured for 6 days under differentiating conditions (B) or kept under proliferating conditions (C) with anti-KLF9 antiserum, anti-KLF13 antiserum or corresponding pre-immune sera (PI). Enrichment of *Gjc2, Mag, Mbp, Plp1* and *Aatk* regulatory regions or control genomic regions (Contr_1, Contr_2) in the immunoprecipitates was determined by qPCR relative to preimmune sera that were arbitrarily set to 1 (red dotted line). Experiments were performed three times with each PCR in triplicate. (**D–F**) Consequences of KLF9 or KLF13 overexpression (oe) on the differentiation of retrovirally transduced wildtype rat (D,E) and KLF13-deficient mouse (F) oligodendroglial cells as analysed by immunocytochemical stainings. (D) Representative staining of differentiating rat cultures after transduction with GFP-, GFP- and KLF9-, or GFP- and KLF13-expressing retroviruses using antibodies directed against GFP and MBP. Scale bar: 50 μm (E, F). Quantification of the percentage (± SEM) of MBP-expressing cells among all oligodendroglial cells transduced with the various retroviruses (*n* = 3, D). Statistical significance (*P ≤ 0.05; **P ≤ 0.01; ***P ≤ 0.001) was determined by one way Anova with Bonferroni correction (A, E, F) or Student's *t* test (B, C).

To be able to directly compare the effects of KLF9 and KLF13 on oligodendrocyte differentiation, we used mouse primary oligodendroglial cultures and treated them with siRNA pools against *Klf9* or *Klf13* and compared the effect with controls. The percentage of MBP-positive cells after transient knockdown of KLF13 in wildtype oligodendroglial cells was comparable to the one observed after KLF9 knockdown and led to similar impairments of oligodendrocyte differentiation as the constitutive KLF13 deletion (compare Figure 11A, left panel with Figure 10A). Whereas knockdown of KLF9 and KLF13 similarly reduced oligodendroglial differentiation capabilities in wildtype cells, knockdown effects in KLF13-deficient cells were different for KLF9 and KLF13 (Figure 11A, right panel). In KLF13-deficient cells, KLF9 knockdown decreased the already impaired differentiation capacity even further. KLF13 knockdown remained without effect. These data strengthen the notion from reporter gene assays that both Klf proteins function similarly in oligodendroglial cells.

In further support of this conclusion, chromatin immunoprecipitation experiments revealed a significant enrichment of regulatory regions from the *Gjc2, Mag, Mbp, Aatk* and *Plp1* genes in chromatin prepared from differentiating rat oligodendroglial cells after precipitation with either anti-KLF9 or anti-KLF13 antibodies as compared to the pre-immune control (Figure 11B). In contrast, other regions in the vicinity of these genes failed to be enriched. We conclude from these findings that both Klf proteins are bound in differentiating oligodendrocytes to regulatory regions of myelin genes. No enrichment of either Klf protein was observed to these regions in chromatin prepared from proliferating oligodendroglial cells arguing that Klf proteins get recruited to these regions during the differentiation process (Figure 11C).

To provide further evidence for a cell-intrinsic redundant function of the Klf proteins in oliogdendroglial cells, we turned from loss-of-function experiments to a gain-of-function approach and asked how ectopic overexpression of KLF9 or KLF13 in retrovirally transduced cells impacts their ability to differentiate. When transduced with a Klf expressing retrovirus, approximately twice as many wildtype oligodendroglial cells expressed MBP after 6 days in differentiating conditions as when transduced with a Gfp-expressing control retrovirus (Figure 11D, E). No substantial differences were observed between KLF9 and KLF13. Intriguingly, both Klf proteins were also equally efficient in rescuing the impaired differentiation capacity of KLF13-deficient mouse oligodendrocytes further supporting their redundant role in oligodendrocyte differentiation and myelination (Figure 11F).

## DISCUSSION

KLF9 has been previously implicated as a regulator of oligodendrocyte differentiation and myelination downstream of T3 (8). In the current study, we obtained deeper mechanistic insights into the role of KLF9. For one, we unravelled its molecular mode of action. We demonstrated that KLF9 recognizes regulatory regions in vitro and in vivo that have been previously shown to bind other transcription factors with roles in oligodendrocyte differentiation such as SOX10 and MYRF (29,34). This included regulatory regions of the *Gjc2, Mag, Mbp, Plp1* and *Aatk* genes. All of them were occupied by KLF9 in oligodendrocytes but not in oligodendrocyte precursors and responded to KLF9 by gene activation in reporter gene assays.

While KLF9 was able to activate these regions to some extent on its own in vitro, it likely requires the cooperation of other transcription factors that are equally bound to these regulatory regions in vivo. This results in a synergistic activation of key differentiation promoting and myelin genes. Similar observations have previously been made for other transcription factors such as Zfp24 (Zfp191) and Nfatc2 (35,36). In case of KLF9, the relevant partners include SOX10 and MYRF so that oligodendrocyte-specific regulatory regions display extremely high activation rates in the combinatorial presence of all three transcription factors. OLIG proteins appear of less significance as cooperation partners of Klf proteins and failed to yield superactivation when additionally combined with SOX10 or MYRF.

While we tested only a limited number of regulatory regions in functional assays, bioinformatic analyses point to a broader relevance of the interaction between KLF9/KLF13 and SOX10. Of 186 regions with SOX10 peaks (29) in or near genes selectively expressed in newly formed oligodendrocytes (30), all but 21 contained at least one potential Klf binding motif in the vicinity of the SOX10 peak (see Materials and Methods). For the 257 regions with SOX10 peaks in or near genes expressed in myelinating oligodendrocytes, the number of regions with at least one Klf binding motif amounted to 227. These data indicate that binding sites for Sox and Klf proteins may be frequently linked in regulatory regions of genes associated with oligodendrocyte differentiation.

We also obtained evidence for a physical interaction with SOX10 in co-immunoprecipitation experiments. GST pulldown experiments confirmed the interaction and pointed to an involvement of the region encompassing the dimerization and DNA-binding HMG-domains in Klf protein recognition. The inability of a SOX10 variant with mutation in the dimerization domain to activate the *Gjc2* promoter synergistically with KLF9 supports the relevance of the physical interaction for the functional cooperation in reporter gene assays.

The contribution of this physical interaction to functional cooperation between KLF9 and SOX10 *in vivo* is less clear and difficult to determine as the consequences of disrupted homodimerization and disturbed interaction with Klf proteins in currently available SOX10 mutants can only be separately assessed on single regulatory regions but not globally. However, the limited impact of the SOX10 dimerization domain mutant on the early steps of oligodendrocyte differentiation in the spinal cord (37) argues that the basis for functional cooperation is likely more complex *in vivo*.

In addition to Klf proteins, SOX10 interacts with MYRF (13) and is capable of recruiting the Mediator complex (38). Intriguingly, we also detected a physical interaction between the Klf proteins and MYRF in co-immunprecipitation experiments. All these interactions may eventually contribute to stabilize a larger enhanceosomal complex on the regulatory regions active in differentiating oligodendrocytes. With each protein having multiple contacts within the complex, loss of single physical interactions may only have mild consequences on enhanceosome formation.

Regarding further aspects of the molecular mode of action of the Klf proteins, all available data indicate that they bind themselves to DNA. The variability of distances and orientation to known or predicted SOX10 binding sites do not favour a model, in which one factor directly facilitates binding of the other. We also do not know whether recruitment of SOX10 and the Klf proteins follows a specific order and whether Klf proteins have additional functions beyond their activity as transcription factors.

The combinatorial mode of gene activation is an easy way for each of the transcription factors to add its unique features to the activation process. KLF9 may for instance contribute T3 effects in this way. However, a combinatorial mode may also mean that most factors are not absolutely essential or even dispensable. This may explain several aspects previously observed for the role of KLF9 and T3 in oligodendrocyte differentiation and myelination (10).

The other intriguing finding of the current study is that KLF9 is largely co-expressed during oligodendroglial development with its close relative KLF13. In contrast, the other two BTEB-subfamily members KLF14 and KLF16 appear to be less relevant in oligodendroglial cells due to low or absent expression. Despite the fact that KLF13 is unresponsive to T3 and cannot mediate T3 effects, both related Klf proteins appear to function in mechanistically similar ways during oligodendrocyte differentiation. They share the ability to bind to oligodendrocyte-specific regulatory regions, interact with SOX10 and MYRF and activate gene expression in synergy with these two transcription factors. Like KLF9, overexpression of KLF13 in oligodendroglial cells increases their differentiation capacity and deficiency decreases it. This functional similarity between KLF9 and KLF13 appears to translate into largely redundant functions during oligodendroglial development. At least partial functional redundancy between KLF9 and KLF13 has also been reported in the nervous system during regulation of clock genes and for hippocampal gene expression (39,40). It likely enables one Klf protein to compensate the loss of the other. This may offer an explanation for previous reports of normal developmental myelination in KLF9-deficient mice (8). It is also consistent with our observation of fairly mild and transient effects on terminal differentiation and myelination in KLF13-deficient mice.

A proof of this assumption would require the generation of mouse mutants with deficiencies of both KLF9 and KLF13. Given the widespread roles and expected redundancies of both proteins, constitutive double-deficient mice are likely not viable. An analysis of the functional redundancy of KLF9 and KLF13 would therefore require a conditional deletion strategy and corresponding floxed alleles. At least for KLF13, such a floxed allele is not yet available.

Loss of KLF9 had previously been reported to impact remyelination after cuprizone-induced demyelination much more strongly than developmental myelination (8). This finding implies that the functional redundancy observed during developmental myelination is no longer in place during remyelination. Therefore, it would be interesting to search in future studies for potential reasons such as divergent expression of KLF9 and KLF13 under remyelination conditions.

Nevertheless the presented data go a long way to characterize the role of the closely related KLF9 and KLF13 in oligodendrocyte development and offer deep insights into their functionally redundant molecular mode of action as synergistic transcriptional activators during terminal differentiation and myelination.

## DATA AVAILABILITY

All data generated or analyzed during this study are included in this published article or deposited in GEO under accession number GSE212736.
